# Freshwater reservoir offsets and food crusts: Isotope, AMS, and lipid analyses of experimental cooking residues

**DOI:** 10.1371/journal.pone.0196407

**Published:** 2018-04-25

**Authors:** John P. Hart, Karine Taché, William A. Lovis

**Affiliations:** 1 Research and Collections Division, New York State Museum, 3140 Cultural Education Center, Albany, United States of America; 2 Department of Anthropology, CUNY Queens College, Queens, United States of America; 3 Department of Anthropology and MSU Museum, Michigan State University, East Lansing, Michigan, United States of America; University of Illinois, UNITED STATES

## Abstract

Freshwater reservoir offsets (FROs) occur when AMS dates on charred, encrusted food residues on pottery predate a pot’s chronological context because of the presence of ancient carbon from aquatic resources such as fish. Research over the past two decades has demonstrated that FROs vary widely within and between water bodies and between fish in those water bodies. Lipid analyses have identified aquatic biomarkers that can be extracted from cooking residues as potential evidence for FROs. However, lacking has been efforts to determine empirically how much fish with FROs needs to be cooked in a pot with other resources to result in significant FRO on encrusted cooking residue and what percentage of fish C in a residue is needed to result in the recovery of aquatic biomarkers. Here we provide preliminary assessments of both issues. Our results indicate that in historically-contingent, high alkalinity environments <20% C from fish may result in a statistically significant FRO, but that biomarkers for aquatic resources may be present in the absence of a significant FRO.

## Introduction

Pottery vessels and fragments thereof are mainstays of archaeological analyses worldwide. Because of uncertain chronological associations between these artifacts and spatially associated charred plant material and animal bone, the ability to directly date these vessels is important. With the development of accelerator mass spectrometry (AMS) radiocarbon dating, the direct dating of charred cooking residues adhering to the interior surfaces of pots and sherds became a common method of obtaining direct age estimates [1,2].

Concern about the accuracy of such age estimates was prominently raised in the early 2000s [3–5]. This concern arose because of the potential for ancient carbon present in freshwater bodies to be metabolized by aquatic organisms and contribute to cooking residue formation when those organisms are subjected to water-based cooking. The presence of ancient carbon in the residues results in radiocarbon ages that are older than the pottery in question. The freshwater reservoir effect (FRE) resulting in freshwater reservoir offsets (FROs) is now well established in the literature [6]. Questions have been raised as to the implication of the FRE in certain site-specific and regional radiocarbon age datasets [7,8] and the use of current hydrological conditions to interpret the past [9]. However, concern remains as to the accuracy of radiocarbon dates obtained on cooking residues, especially when those dates do not match accepted regional chronologies [10–12]. Of primary importance for investigating FROs is understanding how different resources contribute carbon to residue formation, and the variability of ancient carbon sequestered in freshwater bodies and as a result, aquatic organisms both spatially and temporally [13–15].

Experiments with water-based cooking have contributed to understanding how the contributions of C from varying resources affect residue formation [16,17]. Modeling using bulk-stable C isotopes has allowed the estimation of significant FROs with varying raw resource mixes and dead C fractions in aquatic organisms [7,9,18]. The extraction of fatty acids from charred cooking residues and pottery fabric has provided additional evidence for the presence of C from fish in residues. While most published analyses have focused on residues absorbed into the pottery fabric, recent lipid analyses of charred encrusted cooking residues have routinely yielded biomarkers for aquatic resources (e.g., [19–23]). Analyses of contemporary aquatic organisms and the chemistry of freshwater systems have contributed to understandings of spatial and temporal variability in the FRE [13,24]. Empirical investigations of how much fish C in charred residues is needed to produce statistically significant FROs in the presence of FRE and how much fish C is needed for aquatic biomarkers to be identified in residues have been lacking.

In this article, we provide preliminary assessments of both issues. These were accomplished through cooking experiments with proportional mixes of fish and maize. AMS dates were obtained on fish of varying species from three lakes and one stream in New York. We used bulk isotope analyses to assess the proportion of C from fish that contributed to samples obtained from proportionally prepared mixes of dried fish and maize. We used subsamples for radiocarbon dating to determine what proportions of fish C in a residue resulted in significant FROs. We also extracted fatty acids from proportional mixes of maize and fish powders to determine when biomarkers for aquatic resources became evident. Our results provide important new insights into issues surrounding FROs from directly radiocarbon dating charred cooking residues.

## Materials and methods

### Cooking experiments

**Fig 1:**
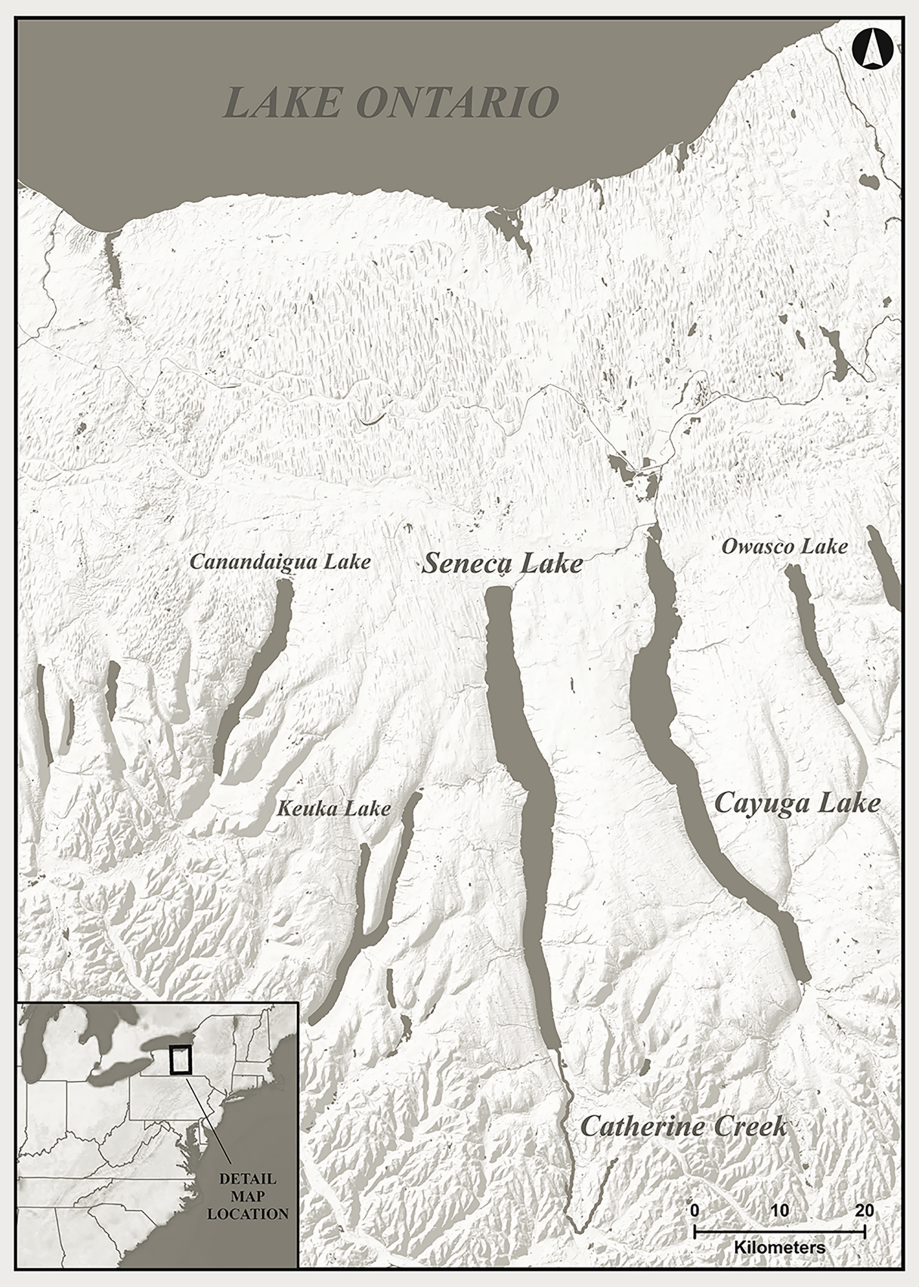
Locations of waterbodies from which fish were captured for the current experiments.

Twenty-three fish and two maize samples were subjected to radiocarbon dating (Table 1). The fish were captured from Lake Ontario in 2014, Seneca Lake in 2015, Cayuga Lake, and Catherine Creek, a tributary of Seneca Lake, in 2016 (Fig 1). The 2014 Lake Ontario Fish was provided dead to the Museum by the New York State Department of Environmental Conservation (NYS DEC). The other fish were obtained by the Museum’s Ichthyology Department during surveys under NYS DEC License to Collect or Possess: Scientific #1809. The fish were euthanized with MS-222 (Tricaine Methanesulfonate) using the methods and concentrations outlined in the 2013 American Veterinary Medical Association's Guidelines for the Euthanasia of Animals.

**Table 1:** ^14^C dating and isotope results for fish from New York freshwater bodies.

UCIAMS	Year	Species	Common Name	Location	δ^13^C^e^	FMC	D^14^C	^14^C age (BP)	FRO^f^	FDC^g^
153202^a^	2014	*Coregonus clupeaformies*	Lake Whitefish	Lake Ontario	-21.9	1.0167±0.0018	8.8±1.8	modern	26±14	0.0000
166335^b^	2015	*Scardinius erythrophthalmus*	Common Rudd	Seneca Lake	-17.2	1.0215±0.0020	21.5±2.0	modern	-7±16	0.0055
166336^b^	2015	*Scardinius erythrophthalmus*	Common Rudd	Seneca Lake	-17.0	1.0264±0.0017	26.4±1.7	modern	-40±13	0.0218
166337^b^	2015	*Scardinius erythrophthalmus*	Common Rudd	Seneca Lake	-16.0	1.0199±0.0016	19.9±1.6	modern	6±12	0.0246
179650	2016	*Oncorhynchus mykiss*	Rainbow Trout	Catherine Creek	-22.0	1.0197±0.0016	19.7±1.6	modern	-12±12	0.0185
179651	2016	*Oncorhynchus mykiss*	Rainbow Trout	Catherine Creek	-19.1	1.0126±0.0012	12.6±1.2	modern	44±9	0.0256
179652^d^	2016	*Salvelinus namaycush*	Lake Trout (1)	Cayuga Lake	-26.5	0.9960±0.0012	-4.0±1.2	30±15	177±10	0.0238
179653	2016	*Salvelinus namaycush*	Lake Trout (2)	Cayuga Lake	-27.7	0.9931±0.0012	-6.9±1.2	55±15	200±9	0.0217
179654^d^	2016	*Salvelinus namaycush*	Lake Trout (3)	Cayuga Lake	-24.2	0.9994±0.0012	-0.6±1.2	5±15	150±9	0.0265
179655^c^	2016	*Salvelinus namaycush*	Lake Trout (4)	Cayuga Lake	-24.9	0.9922±0.0012	-7.8±1.2	65±15	208±10	0.0224
179656^d^	2016	*Salvelinus namaycush*	Lake Trout (5)	Cayuga Lake	-26.8	0.9939±0.0013	-6.1±1.3	50±15	194±11	0.0209
179657^d^	2016	*Micropterus salmoides*	Largemouth Bass	Cayuga Lake	-23.7	0.9967±0.0013	-3.9±1.3	30±15	176±10	0.0275
179658	2016	*Salmo salar*	Atlantic Salmon	Cayuga Lake	-27.5	0.9913±0.0012	-8.7±1.2	70±15	215±10	0.028
179659	2016	*Salmo salar*	Atlantic Salmon	Cayuga Lake	-25.2	0.9954±0.0012	-4.6±1.2	35±15	182±9	0.0059
179660	2016	*Salmo salar*	Atlantic Salmon	Cayuga Lake	-24.1	0.9969±0.0012	-3.1±1.2	25±15	170±9	0.0000
179661	2016	*Salmo salar*	Atlantic Salmon	Cayuga Lake	-24.9	0.9902±0.0012	-9.8±1.2	80±15	224±10	0.0074
179662^c^	2016	*Esox niger*	Chain Pickerel	Cayuga Lake	-20.9	0.9897±0.0013	-10.3±1.3	85±15	228±11	0.0111
179663	2016	*Oncorhynchus mykiss*	Rainbow Trout	Catherine Creek	-19.2	1.0122±0.0012	12.2±1.2	modern	47±9	0.006
179664	2016	*Catostomus commersonii*	White Sucker	Catherine Creek	-21.9	1.0288±0.0013	28.8±1.3	modern	-83±10	0.0123
179665	2016	*Catostomus commersonii*	White Sucker	Catherine Creek	-20.6	1.0107±0.0013	10.7±1.3	modern	60±10	0.0000
179666	2016	*Catostomus commersonii*	White Sucker	Catherine Creek	-21.8	1.0069±0.0012	6.9±1.2	modern	90±10	0.0055
179667	2016	*Catostomus commersonii*	White Sucker	Catherine Creek	-23.6	1.0121±0.0012	12.1±1.2	modern	49±10	0.0218
179668	2016	*Catostomus commersonii*	White Sucker	Catherine Creek	-21.5	1.0057±0.0012	5.7±1.2	modern	99±10	0.0246
166338	2015	*Zea mays* ssp. *mays*	Maize	Washington Co.	-11.9	1.0205±0.0016	20.6±1.6	modern	0	0
180884	2016	*Zea mays* ssp. *mays*	Maize	Washington Co.	-11.9	1.0181±0.0016	18.2±1.6	modern	0	0

^a^Used in 2014 proportional cooking experiments with maize meal.

^b^Used in 2015 proportional cooking experiments with maize meal.

^c^Used in 2016 proportional powder mixes with maize.

^d^Used in 2016 proportional cooking experiments with maize kernels.

^e^Measured to a precision <0.1‰.

^f^Equations from [13]

FRO = ‒8033*ln(FMC_sample_/FMC_atmosphere_)

FRO 1σ = ‒8033*ln((FMC_sample_)+(FMC_1σ_))+(8033*ln(FMC_sample_))

^g^FDC = (FMC_maize_- FMC_sample_)/FMC_maize._ Negative values round to 0

All fish were kept frozen until muscle tissue was sampled. The sampled muscle tissue was freeze dried before submission for AMS dating. Commercial cornmeal was used in the 2014 cooking experiments. Ears of Dent maize (*Zea mays* ssp. *mays*) were obtained from an organic Amish farm in northern Washington County, New York USA for the 2015 and 2016 cooking experiments. Maize samples from 2015 and 2016 were submitted for AMS dating. The ears were allowed to completely dry in an unheated herbarium drying cabinet. Dried kernels were ground to meal. Whole kernels were rehydrated by soaking in ultra-purified water for ~16 hours. Whole kernel and meal were both used in water-based cooking in northeastern North America [25,26]. Raw fish muscle tissue from thawed fish with relatively large FROs (Table 1) was divided into 2.5g pieces, which were kept refrigerated until used in cooking experiments.

Forty-five 25g resource mixes using whole kernels or meal with fish muscle tissue (Table 2) were prepared in 10% or 20% increments and placed in 400 ml of ultra-purified water in Pyrex beakers. The beakers were placed on a hot plate at 400^°^C and boiled for 1 hr. At 1 hr the material in solution and suspended in the liquid was sampled by pipette (whole kernel) or by decanting (meal) into glass test tubes (hereafter, “liquid” samples). Material adhering to the interior beaker wall was scraped off from the water line to the lip of the beaker and placed in a glass test tube containing ultra-purified water (hereafter, “wall” samples). All samples were frozen and then freeze dried. Freeze-dried samples were kept in a desiccation chamber until sampled for analyses.

**Table 2:** Percent Fish C in residues from all cooking experiments.

Lab No.	Fish	Maize Form	Source	% Raw Fish	Residue δ ^13^C	% Fish C in residue^a^
UCIAMS 185316	Lake Trout 5	kernel	liquid	90	-22.9	70.38
UCIAMS 185315	Lake Trout 3	kernel	liquid	80	-21.5	65.67
UCIAMS 185314	Lake Trout 3	kernel	liquid	70	-20.0	55.31
UCIAMS 185313	Lake Trout 3	kernel	liquid	60	-19.6	20.06
UCIAMS 185312	Lake Trout 3	kernel	liquid	50	-17.5	38.62
UCIAMS 185311	Lake Trout 3	kernel	liquid	40	-17.6	38.74
UCIAMS 185310	Lake Trout 3	kernel	liquid	30	-15.2	22.71
UCIAMS 185309	Lake Trout 3	kernel	liquid	20	-13.8	12.96
UCIAMS 185308	Lake Trout 5	kernel	liquid	10	-13.2	8.12
UCIAMS 185325	Largemouth Bass	kernel	liquid	90	-22.9	92.92
UCIAMS 185324	Largemouth Bass	kernel	liquid	80	-20.2	69.76
UCIAMS 185323	Largemouth Bass	kernel	liquid	70	-19.3	62.75
UCIAMS 185322	Largemouth Bass	kernel	liquid	60	-17.5	47.55
UCIAMS 185321	Largemouth Bass	kernel	liquid	50	-17.4	46.79
UCIAMS 185320	Largemouth Bass	kernel	liquid	40	-16.4	37.68
UCIAMS 185319	Largemouth Bass	kernel	liquid	30	-15.1	27.21
UCIAMS 185318	Largemouth Bass	kernel	liquid	20	-17.4	46.11
UCIAMS 185317	Largemouth Bass	kernel	liquid	10	-13.9	16.90
UCB ISO-17-04_C5	Lake Trout 5	kernel	wall	90	-25.4	92.60
UCB ISO-17-04 rerun_A3	Lake Trout 3	kernel	wall	80	-26.0	96.40
UCB ISO-17-04_A6	Lake Trout 3	kernel	wall	70	-25.5	93.25
UCB ISO-17-04_C2	Lake Trout 3	kernel	wall	60	-24.8	84.83
UCB ISO-17-04_B1	Lake Trout 3	kernel	wall	50	-24.3	73.77
UCB ISO-17-04_C4	Lake Trout 3	kernel	wall	40	-22.7	76.75
UCB ISO-17-04_A9	Lake Trout 3	kernel	wall	30	-23.1	60.18
UCB ISO-17-04_C12	Lake Trout 3	kernel	wall	20	-20.7	90.96
UCB ISO-17-04_B5	Lake Trout 5	kernel	wall	10	-25.2	92.60
UCB ISO-17-04_A2	Largemouth Bass	kernel	wall	90	-24.0	102.55
UCB ISO-17-04_C9	Largemouth Bass	kernel	wall	80	-23.5	97.63
UCB ISO-17-04_A10	Largemouth Bass	kernel	wall	70	-23.6	98.93
UCB ISO-17-04_A4	Largemouth Bass	kernel	wall	60	-23.1	94.27
UCB ISO-17-04_A3	Largemouth Bass	kernel	wall	50	-22.3	87.53
UCB ISO-17-04_A11	Largemouth Bass	kernel	wall	40	-22.2	86.76
UCB ISO-17-04_A5	Largemouth Bass	kernel	wall	30	-21.1	77.56
UCB ISO-17-04_B10	Largemouth Bass	kernel	wall	20	-23.7	92.14
UCB ISO-17-04_B4	Largemouth Bass	kernel	wall	10	-22.8	102.55
UCIAMS 166316	Common Rudd 1	meal	liquid	90	-13.3	27.11
UCIAMS 166317	Common Rudd 1	meal	liquid	70	-12.1	4.85
UCIAMS 166318	Common Rudd 1	meal	liquid	50	-12.1	4.45
UCIAMS 166319	Common Rudd 1	meal	liquid	30	-12.1	3.55
UCIAMS 166320	Common Rudd 1	meal	liquid	10	12.0	2.28
UCIAMS 166321	Common Rudd 2	meal	liquid	90	-13.2	26.99
UCIAMS 166322	Common Rudd 2	meal	liquid	80	-12.6	14.86
UCIAMS 166323	Common Rudd 2	meal	liquid	70	-12.3	8.73
UCIAMS 166324	Common Rudd 2	meal	liquid	60	-12.2	6.00
UCIAMS 166325	Common Rudd 2	meal	liquid	50	-12.0	2.27
UCIAMS 166326	Common Rudd 2	meal	liquid	40	-12.1	4.03
UCIAMS 166327	Common Rudd 2	meal	liquid	30	-12.1	3.85
UCIAMS 166328	Common Rudd 2	meal	liquid	20	-12.1	3.88
UCIAMS 166329	Common Rudd 2	meal	liquid	10	-11.9	0.00
UCIAMS 166330	Common Rudd 3	meal	liquid	90	-13.3	35.36
UCIAMS 166331	Common Rudd 3	meal	liquid	70	-12.2	7.69
UCIAMS 166332	Common Rudd 3	meal	liquid	50	-12.1	5.39
UCIAMS 166333	Common Rudd 3	meal	liquid	30	-12.1	5.65
UCIAMS 166334	Common Rudd 3	meal	liquid	10	-11.8	0.00
UCB ISO-16-03_Tray2_C2	Common Rudd 2	meal	wall	90	-13.3	27.13
UCB ISO-16-03_Tray2_C4	Common Rudd 2	meal	wall	80	-13.7	35.55
UCB ISO-16-03_Tray2_C3	Common Rudd 2	meal	wall	70	-13.0	21.79
UCB ISO-16-03_Tray2_B10	Common Rudd 2	meal	wall	60	-12.3	7.62
UCB ISO-16-03_Tray2_B8	Common Rudd 2	meal	wall	50	-12.2	5.60
UCB ISO-16-03_Tray2_C1	Common Rudd 2	meal	wall	40	-12.5	11.57
UCB ISO-16-03_Tray2_B9	Common Rudd 2	meal	wall	30	-12.3	7.68
UCB ISO-16-03_Tray2_B11	Common Rudd 2	meal	wall	20	-12.1	3.08
UCB ISO-16-03_Tray2_B12	Common Rudd 2	meal	wall	10	-12.4	10.16
UCIAMS 153203	Whitefish	meal	wall	80	-15.3	39.27
UCIAMS 153204	Whitefish	meal	wall	70	-12.4	12.58
UCIAMS 153205	Whitefish	meal	wall	60	-14.2	29.12
UCIAMS 153206	Whitefish	meal	wall	50	-14.5	32.15
UCIAMS 153207	Whitefish	meal	wall	40	-13.1	19.71
UCIAMS 153208	Whitefish	meal	wall	30	-12.0	9.36
UCIAMS 153209	Whitefish	meal	wall	20	-11.5	4.34
UCIAMS 153210	Whitefish	meal	wall	10	-10.9	0.00

^a^Mass Balance:(δ^13^C_sample_ ‒ δ^13^C_maize_)/(δ^13^C_fish_ ‒ δ^13^C_maize_))*100

### Resource powder mixes

Freeze-dried Lake Trout and Chain Pickerel muscle tissue and dried whole maize kernels were ground into 0.5 mm powders. These were mixed in 10% increments in 1g samples (10% and 90% maize to 90% fish and 10% maize by weight). Each thoroughly mixed sample (~0.025 g) was subsampled for AMS dating, and the remainder was subjected to lipid analyses.

### Isotope analysis and AMS dating

All samples for AMS dating were submitted to the W. M. Keck Carbon Cycle Accelerator Mass Spectrometry Laboratory at the University of California-Irvine. These included samples from the 23 fish, two maize kernels, 18 cooking experiments, and 18 resource powder mixes. Protocols for AMS dating modern samples are documented on the laboratory’s website (https://www.ess.uci.edu/group/ams/home). Stable carbon isotope assays (δ^13^C) were performed on samples at the Keck laboratory or the University of California Berkeley’s Center for Stable Isotope Biogeochemistry.

### Lipid analysis of powder mixes

Fish-maize powder mixes were subjected to lipid analysis to determine when biomarkers for aquatic resources become evident. Liquid samples obtained after the cooking experiment were also analyzed but lipid yields obtained were too low to be interpretable (<0.5μg/mg of lipids per mg of residue sample). As described above, the samples consisted of freeze-dried fish muscle tissue and dried whole maize kernels ground into 0.5 mm powders. These were mixed in 10-percent increments in 1 g samples (10% and 90% maize to 10% fish and 90% maize by weight). To generate ω-(o-alkylphenyl)alkanoic acids, sterile clay powder was added to the fish-maize mixes [27,28]. Clay powder was obtained by drilling and reducing to powder 20 grams of a replica vessel using a Dremel^TM^ tool and placing the resulting clay powder in a furnace at 500°C for 6 hours to completely remove any organic material in the clay. Nine samples consisting of 1 gram of sterile ceramic powder mixed with different proportions of dried Chain Pickerel and maize were placed in sealed hach tubes in a furnace at 270°C for 17 hr, following previous experiments which established these conditions as prerequisite to the formation of ω-(o-alkylphenyl)alkanoic acids [28]. Three samples consisting of single ingredients (i.e., dried maize, dried Chain Pickerel, and dried Lake Trout) mixed with sterile clay powder were also exposed to intense heating in sealed hach tubes (270°C for 17 hours). Nine samples consisting of 1 gram of sterile ceramic powder mixed with different proportions of Lake Trout and maize were analyzed without being heated to assess the nature and quantity of aquatic biomarkers detected when resources are not subjected to intense heating.

Lipids were extracted from the 21 samples by direct methylation with acidified methanol to maximize recovery [29]. Methanol (4mL) was added and homogenized with the ceramic-fish-maize samples. Each mixture was ultra-sonicated for 15 minutes and then acidified with concentrated sulphuric acid (800*μ*L). The acidified suspension was heated in sealed tubes for four hours at 70°C and then cooled, and lipids were extracted with n-hexane (3×2mL). Lipids extracted from ceramic matrices were analyzed by gas chromatography–mass spectrometry (GC-MS), a technique that allows the separation of complex mixtures and the identification of plant- and animal-derived lipids. GC-MS analysis was performed using an Agilent 7890A Series gas chromatograph connected to an Agilent 5975 C Inert XL mass-selective detector with a quadrupole mass analyzer (Agilent Technologies, Cheadle, Cheshire, UK). The splitless injector and interface were maintained at 300^°^C and 280^°^C respectively. The carrier gas used was helium at a constant flow of 3ml/min, and the initial inlet/column head pressure was 24.012 psi. The GC column was inserted directly into the ion source of the mass spectrometer. The ionization energy was 70 eV and spectra were obtained by scanning between m/z 50 and 800. A DB-5ms (5%-phenyl)-methylpolysiloxane column (30 m x 0.25mm x 0.25μm; J&W Scientific, Folsom, CA, USA) was used for scanning and SIM. Two distinct runs in SIM mode were conducted. In the first run, a group of ions (*m/z* 74, 105, 262, 290, 318, 346) corresponding to *ω*-(*o*-alkylphenyl) alkanoic acids of carbon length C_16_ to C_22_ were monitored. In the second run, a first group of ions (*m/z* 74, 87, 213, 270) corresponding to 4,8,12-trimethyltridecanoic acid (TMTD) fragmentation, a second group of ions (*m/z* 74, 88, 101, 312) corresponding to pristanic acid, and a third group of ions (*m/z* 74, 101, 171, 326) corresponding to phytanic acid were monitored, respectively.The temperature program was 2 min at 50^°^C, 10^°^C min^–1^ to 325^°^C and 15 min at 325^°^C. The same chromatographic conditions were used in scanning and SIM mode.

## Results

### AMS dates on fish

Results of the AMS dating of the fish samples are presented in Table 1. FROs and their standard deviations were calculated using the formulae in [13]. Fraction of modern carbon (FMC) measures on maize were used in the FRO formula for the fish caught in 2015 and 2016. For the fish caught in 2014, a Northern Hemisphere atmospheric value of 1.020 was used [30].

Twelve of the samples returned modern ages. FROs for four of these ages are negative, while the others range from 6±12 to 99±10 ^14^Cyr. Fish obtained from Cayuga Lake during September of 2016 were the only ones to produce non-modern ^14^C ages. The largest offsets for these fish ranged from 150±9 to 228±11 ^14^Cyr. In total, the variation in FROs on fish were consistent with results obtained in other parts of the world, but which can range to >1,000 ^14^Cyr [11,13,24] depending on the species of fish and the water body’s historically contingent total alkalinity. Oversaturation of carbonate (CO_3_^‒2^) and bicarbonate (HCO_3_^‒^) ions has been documented in Lake Cayuga through sediment analyses to have occurred during the historical and modern periods but not during all portions of the Holocene [31]. Measures of total alkalinity for this lake in September 2016 ranged from 93.8 to 114 mg CaCO_3_/L [32], consistent with expectations for the presence of FROs [9].

### Contribution of fish C to residues

Previous experiments have investigated the relationship of resource mixes to bulk δ^13^C values on residues to determine if those values can be used to detect if maize was cooked in a given pot [7,17,33]. These experiments involved proportional mixes of maize, a C_4_ plant, with C_3_-resources, including wild rice (*Zizania* sp.), chenopodium (*Chenopodium album*), and C_3_-plant consuming white tailed deer (*Odocoileus virginianus*). While it was determined that it is possible to track changes in maize use through time using bulk δ^13^C values in some regions [34,35], it is not possible to use those values independently to determine if maize was cooked or processed in a specific pot [7,17,31] contrary to earlier suggestions [36,37].

Mass balance using δ^13^C values on charred residues cannot be used to determine the percentage of raw maize cooked in a pot as suggested by Morton and Schwarcz [33]. Rather, it indicates the contribution of maize C to residue formation, which itself is determined by C from maize and the resources it was cooked with entering into suspension and solution and being deposited and burned on pottery surfaces [7]. The amount of C from maize and other resources in suspension and solution depends on both boiling time and the form of maize being cooked, as well as the percentage of C in the respective resources [17].

The mass balance formula was used here with δ^13^C and FMC values to determine the percent of fish C in the 72 experimental residues and 18 fish-maize powder mixtures (Tables 2–4). Experiments suggest that heating has little effect on boiled bulk fish flesh δ^13^C (<0.5‰) values [38], and that charring has little effect on grain (<1.0‰) δ^13^C values [39].

**Table 3:** Fish and maize kernel liquid sample residue data.

UCIAMS#	% Raw Fish	δ^13^C (‰)	FMC	D^14^C (‰)	^14^C age (BP)	FRO	% Fish C δ^13^C^a^	% Fish C FMC^b^	FDC
Largemouth Bass									
179657	100	-23.7±.01	0.9967±0.0013	-3.9±1.3	30±15	176±10	100	100	0.0275
185325	90	-22.9±.01	0.9962±0.0012	-3.8±1.2	30±15	176±10	92.92	99.05	0.0216
185324	80	-20.2±.01	0.9980±0.0016	-2.0±1.6	15±15	161±13	69.76	90.66	0.0198
185323	70	-19.3±.01	1.0015±0.0013	1.5±1.3	>Modern	133±10	62.75	75.06	0.0164
185322	60	-17.5±.01	1.0042±0.0013	4.2±1.3	>Modern	111±10	47.55	62.81	0.0137
185321	50	-17.4±.01	1.0043±0.0013	4.3±1.3	>Modern	110±10	46.79	62.52	0.0137
185320	40	-16.4±.01	1.0077±0.0013	7.7±1.3	>Modern	83±10	37.68	47.02	0.1030
185319	30	-15.1±.01	1.0084±0.0014	8.4±1.4	>Modern	78±11	27.21	44.11	0.0096
185318	20	-17.4±.01	1.0016±0.0019	1.6±1.9	>Modern	132±15	46.11	74.67	0.0163
185317	10	-13.9±.01	1.0130±0.0016	13.0±1.6	>Modern	41±12	16.90	23.50	0.0051
180884	0	-11.9±.01	1.0182±0.0016	18.2±1.6	>Modern	0	0	0	0
Lake Trout									
179654^c^	100	-24.2±.01	0.9994±0.0012	-0.6±1.2	5±15	150±9	100	100	0.0265
179656^d^	100	-26.8±.01	0.9939±0.0013	-6.1±1.3	50±15	194±11	100	100	0.0209
185316 ^d^	90	-22.9±.01	0.9996±0.0013	-0.4±1.3	5±15	148±10	70.38	83.49	0.0182
185315^c^	80	-21.5±.01	1.0007±0.0013	0.7±1.3	0±15	139±10	65.67	78.54	0.0172
185314^c^	70	-20.0±.01	1.0031±0.0013	3.1±1.3	>Modern	120±10	55.31	67.88	0.0148
185313^c^	60	-19.6±.01	0.9932±0.0013	-6.8±.1.3	55±15	200±10	20.06	112.57	0.0246
185312^c^	50	-17.5±.01	1.0074±0.0013	7.4±1.3	>Modern	86±10	38.62	48.77	0.0107
185311^c^	40	-17.6±.01	1.0081±0.0013	8.1±1.3	>Modern	80±11	38.74	45.33	0.0099
185310^c^	30	-15.2±.01	1.0110±0.0013	11.0±1.3	>Modern	57±10	22.71	32.14	0.0070
185309^c^	20	-13.8±.01	1.0160±0.0013	16.0±1.3	>Modern	17±10	12.96	9.68	0.0021
185308^d^	10	-13.2±.01	1.0159±0.0013	15.9±1.3	>Modern	18±11	8.12	10.40	0.0023
180884	0	-11.9±.01	1.0182±0.0016	18.2±1.6	>Modern	0	0	0	0

Mass balance: ^a^(δ^13^C_sample_ ‒ δ^13^C_maize_)/(δ^13^C_fish_ ‒ δ^13^C_maize_))*100

^b^(FMC_sample_ ‒ FMC_maize_)/(FMC_fish_ ‒ FMC_maize_))*100

^c^Lake Trout 3. ^d^Lake Trout 5.

**Table 4:** Fish and maize powder mix data.

UCIAMS#	% Raw Fish	δ^13^C (‰)	FMC	D^14^C (‰)	^14^C age (BP)	FRO	% Fish C δ^13^C^a^	% Fish C FMC^b^	FDC
Chain Pickerel									
179662	100	-20.9±.01	0.9897±0.0013	-10.3±1.3	85±15	228±11	100	100	0.0280
180867	90	-20.1±.01	0.9904±0.0016	-9.6±1.6	75±15	222±13	91.64	97.64	0.0273
180868	80	-20.0±.01	0.9938±0.0017	-6.2±1.7	50±15	195±14	90.39	85.77	0.0240
180996	70	-18.8±.01	0.9982±0.0016	-1.8±1.6	15±15	160±13	77.25	70.41	0.0197
180869	60	-18.3±.01	0.9972±0.0016	-2.8±1.6	25±15	167±13	70.87	73.78	0.0206
180870	50	-17.2±.01	1.0018±0.0020	1.8±2.0	modern	130±16	59.15	57.65	0.0161
180871	40	-17.0±.01	0.9988±0.0017	-1.2±1.7	10±15	154±14	56.61	68.145	0.0191
180872	30	-15.0±.01	1.0084±0.0016	8.4±1.6	modern	77±13	34.83	34.31	0.0096
180873	20	-15.1±.01	1.0092±0.0018	9.2±1.8	modern	71±15	35.80	31.60	0.0088
180874	10	-13.0±.01	1.0154±0.0016	15.4±1.6	modern	22±13	12.65	9.75	0.0027
180884	0	-11.9±.01	1.0182±0.0016	18.2±1.6	modern	0	0	0	0
Lake Trout									
179656	100	-24.9±.01	0.9922±0.0012	-7.8±1.2	65±15	208±10	100	100	0.0255
180875	90	-24.4±.01	0.9925±0.0019	-7.5±1.9	60±20	205±16	96.30	98.67	0.0252
180876	80	-23.6±.01	0.9923±0.0016	-7.7±1.6	60±15	207±13	90.06	99.63	0.0254
180877	70	-22.3±.01	0.9984±0.0016	-1.6±1.6	15±15	158±13	80.03	76.19	0.0195
180878	60	-21.3±.01	0.9991±0.0018	-0.9±1.8	5±15	152±15	71.96	73.46	0.0188
180879	50	-19.4±.01	1.0036±0.0017	3.6±1.7	modern	116±14	57.73	55.96	0.0143
180880	40	-18.0±.01	1.0065±0.0016	6.5±1.6	modern	93±13	46.61	45.05	0.0115
180881	30	-16.7±.01	1.0082±0.0016	8.2±1.6	modern	79±13	36.96	38.39	0.0098
180882	20	-18.1±.01	1.0032±0.0016	3.2±1.6	modern	119±13	47.36	57.58	0.0147
180883	10	-14.2±.01	1.0135±0.0016	13.5±1.6	modern	37±13	17.35	17.96	0.0046
180884	0	-11.9±.01	1.0182±0.0016	18.2±1.6	modern	0	0	0	0

Mass balance: ^a^(δ^13^C_sample_ ‒ δ^13^C_maize_)/(δ^13^C_fish_ ‒ δ^13^C_maize_))*100

^b^(FMC_sample_ ‒ FMC_maize_)/(FMC_fish_ ‒ FMC_maize_))*100.

**Fig 2:**
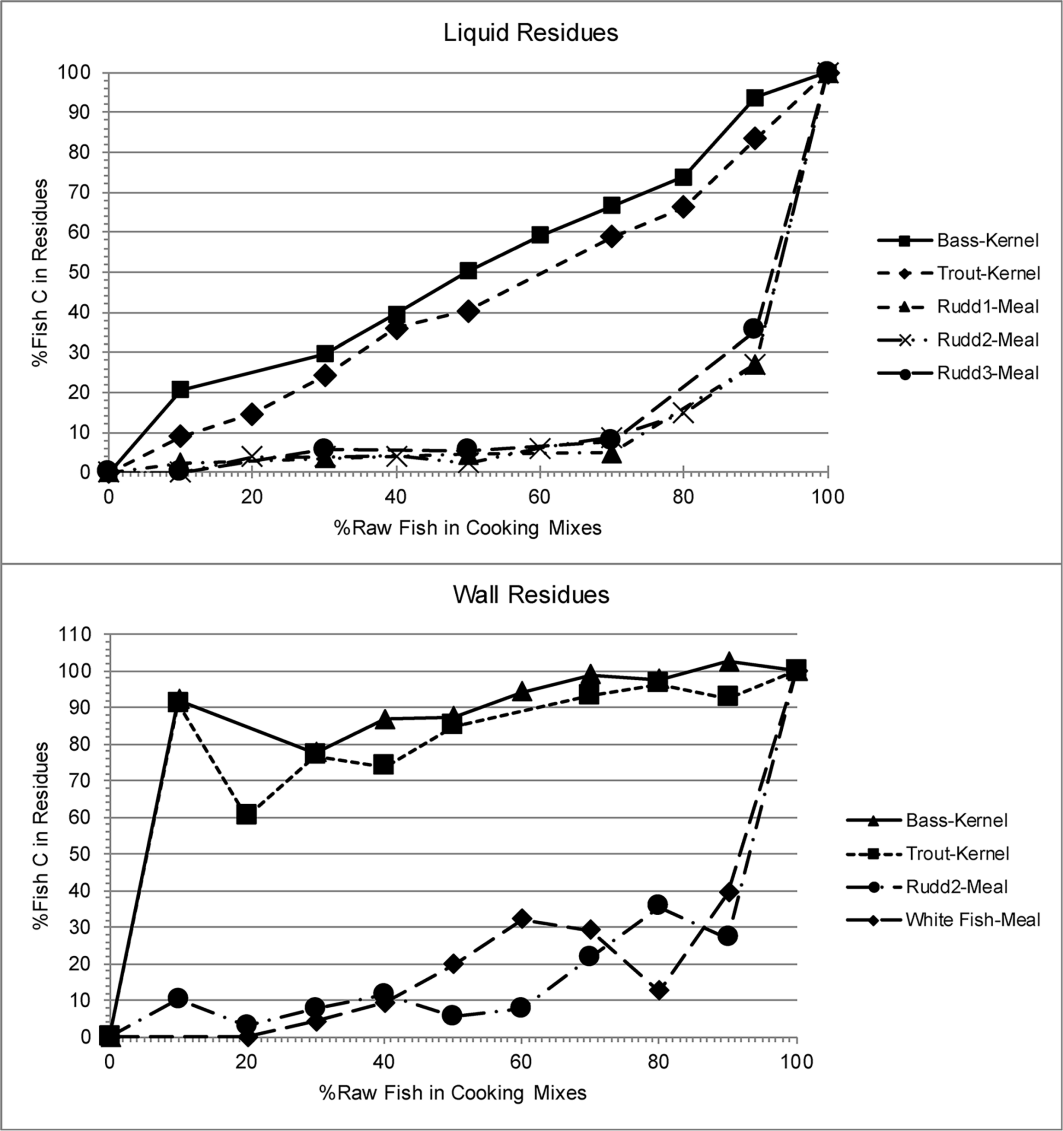
Plots of % raw fish vs. % fish C in residues based on mass balance using δ^13^C values.

Consistent with previous results [7,17], the form of maize and the origination of the sample affected the percent fish C that contributed to the residues (Table 5). Fish C in the wall scraped residues was overrepresented relative to raw resources percentages when cooked with whole kernels. It was underrepresented relative to raw resource percentages when cooked with meal. In the liquid samples, there was an almost linear relationship between the percent raw fish and fish C in the residue when fish was cooked with whole kernels (Fig 2). When cooked with meal, fish C was underrepresented in the liquid sample residues.

**Table 5:** Percent fish C in experimental residues calculated with mass balance from δ^13^C values.

Fish	Maize Form	Sample	Percent Raw Fish (wt)								
			10	20	30	40	50	60	70	80	90
Largemouth Bass	Kernel	Liquid	20.61	43.23	29.63	39.45	50.31	59.22	66.67	73.82	93.73
Lake Trout	Kernel	Liquid	8.92	14.57	24.12	39.96	40.25	53.82	58.89	66.27	83.30
Common Rudd 1	Meal	Liquid	2.28	—	3.55	—	4.45	—	4.85	—	27.11
Common Rudd 2	Meal	Liquid	0.00	3.88	3.85	4.03	2.27	6.00	8.73	14.86	26.99
Common Rudd 3	Meal	Liquid	0.00	—	5.65	—	5.39	—	7.69	—	35.36
Largemouth Bass	Kernel	Wall	92.14	99.50	77.56	86.76	87.53	94.27	98.93	97.63	100.00
Lake Trout	Kernel	Wall	90.96	60.18	76.75	73.77	84.83	88.30	93.25	96.40	92.10
Common Rudd 2	Meal	Wall	10.16	3.089	7.68	11.57	5.60	7.62	21.79	33.55	27.13
Lake Whitefish	Meal	Wall	—	0.00	4.34	9.36	19.71	32.15	29.12	12.58	39.27

### AMS dates and FROs

AMS dates were obtained on the 18 liquid residue samples and 18 maize-fish powder mixes to assess when the contribution of fish C to residue formation may result in significant FROs (Tables 3 and 4). Two of the liquid residue age estimates (UCI 185313, 185318) were not used in the analyses because they produced ages that were substantially older than expected based on their position in the proportional mix sequences. We assume that old carbon was introduced to the samples at some point in processing.

**Fig 3:**
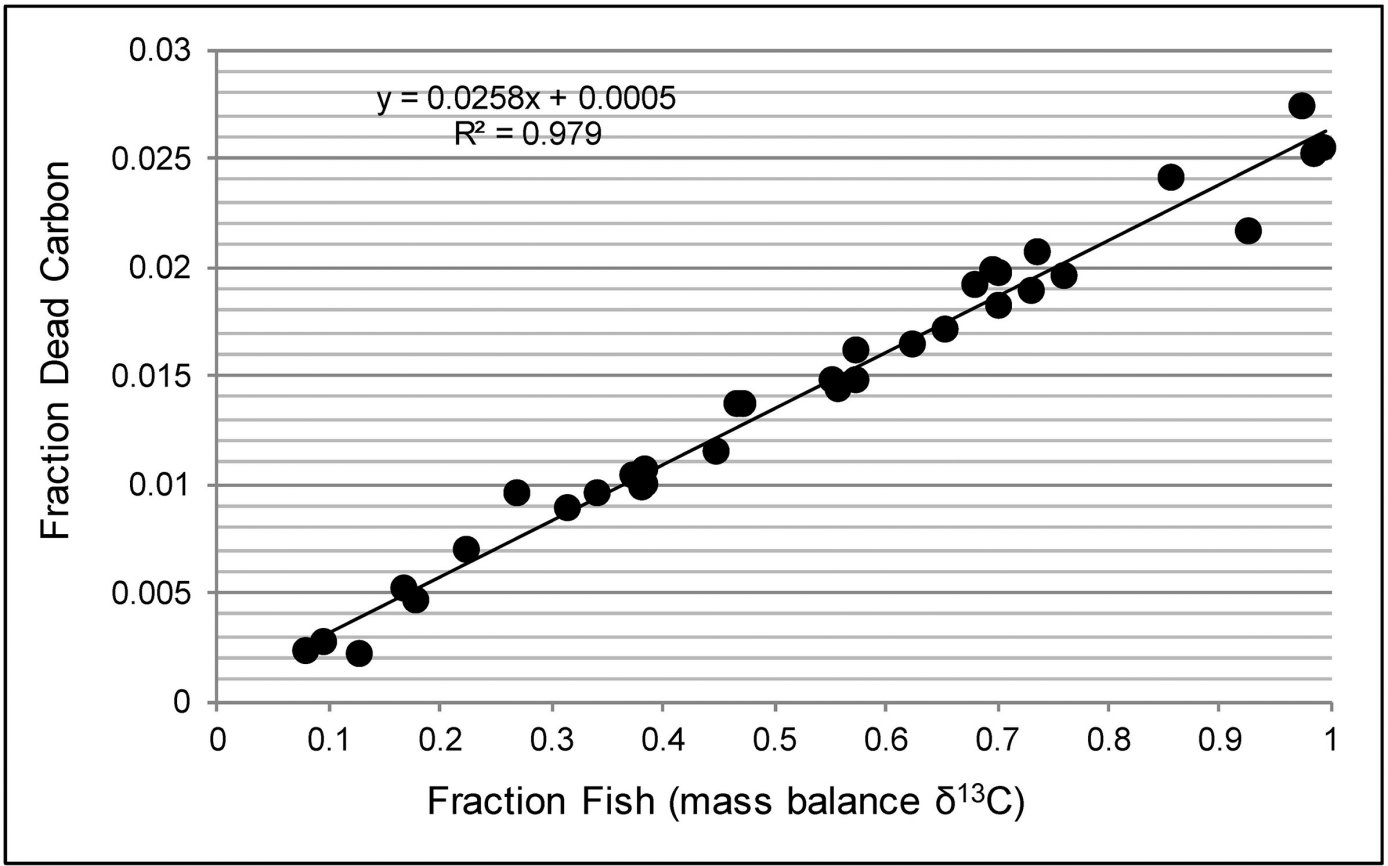
Regression of fraction fish C on fraction dead carbon.

**Fig 4:**
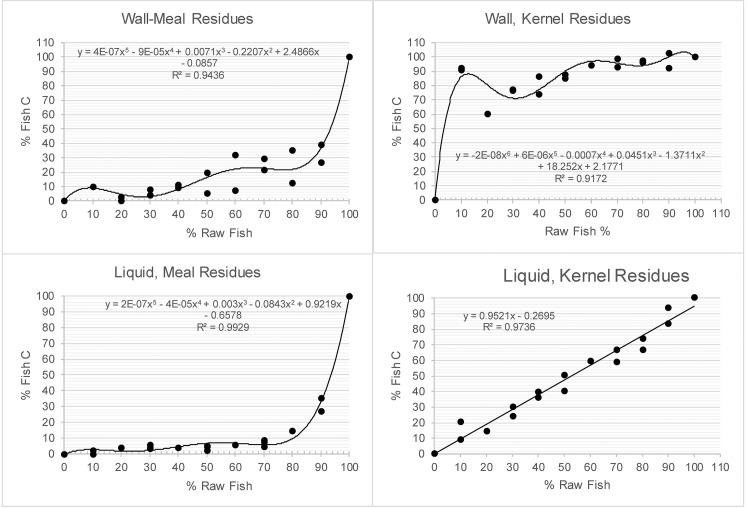
Regressions of % raw fish on % fish C in residues.

There was a very high positive correlation (r = 0.989) between the fraction of fish C in the residues and Fraction Dead Carbon (FDC; Fig 3), and because there was a very high positive correlation between FDC and FRO, there was the same very high positive correlation between the percentage of fish C in residues and FRO. Ward and Wilson’s [40] test was used to determine significant FROs at varying error terms reported with radiocarbon dates. These results were used to calculate the FDC needed to result in statistically significant FROs. The least-squares regression formula in Fig 3 was used to determine the percentage fish C contributing to residue formation needed to result in the calculated FDC for the FROs. Least squares regression was performed for the fish and kernel liquid residues and fifth- and sixth-order polynomial regressions were performed to best fit for the other residues to determine what percentage of raw fish contributed sufficient C in the residues to result in the FDC to produce statistically significant FROs (Table 6, Fig 4).

**Table 6:** Percent raw fish resulting in significant FRO with different radiocarbon date error terms.

Error (±yr)	Significant FRO (yr)	FDC	% Residue Fish C	Percent Raw Fish (wt)			
				Wall-Meal	Wall-Kernel	Liquid-Meal	Liquid-Kernel
15	42	0.00519	18.18	46.33	0.94	46.62	19.38
20	56	0.00690	28.84	55.74	1.66	55.03	30.58
35	97	0.01190	44.19	78.27	2.86	64.56	46.70
50	139	0.01705	64.15	88.72	4.97	73.37	67.65
65	181	0.02220	84.20	93.11	9.07	79.42	88.74

As is evident from the data in Table 6, there was no single relationship between the amount of raw fish in a cooking mixture and a statistically significant FRO as calculated with [40]. This was due to the amount of C in any given fish, and the form of the maize in the mix. This can be understood as follows:

- For wall scraped samples an age estimate with a typical 15-yr error term requires 18.18% fish C in the residue. This is realized when there is between 0.94% and 46.33% raw fish content in the mix for wall-scraped residues dependent on maize in whole kernel and meal form, respectively.
- Wall scraped samples with a less-typical 65-yr error require 84.20% fish C in a residue. Depending on the form of maize in the mix this is realized with between 9.07% and 91.11% raw fish in the mix, respectively.

Results for the liquid-derived samples, representing potential C contribution to residue formation, can be understood as follows:

- For a 15-yr error, the mix would require between 46.62% and 19.38% raw fish for maize in meal and kernel form, respectively, to result in a statistically significant FRO.
- For a 65-yr error term, the mix would require from 79.42% to 88.74% raw fish, with maize meal and kernels, respectively, to result in a statistically significant FRO.

These results demonstrate that it is possible for resource mixes including very little fish C, as low as 0.94%, to result in a statistically significant FRO depending on the form of the other resources in the mix and the radiocarbon date error term. It is also possible that fish must constitute the bulk of the resources being cooked depending on the same variables.

### Lipid analysis and the identification of aquatic biomarkers in fish-maize powder mixes

**Fig 5:**
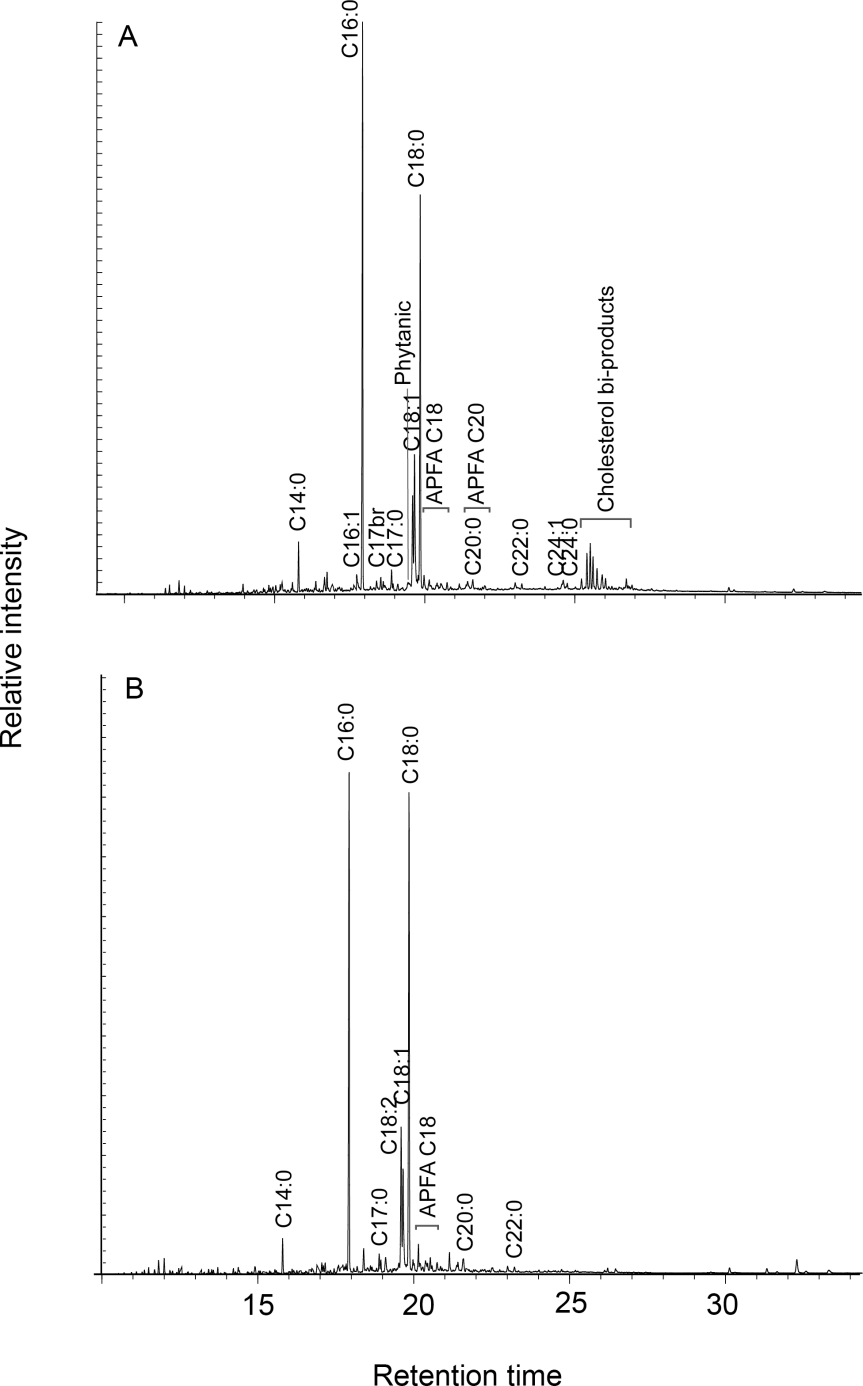
Gas chromatograms of lipid extracts from unheated maize-fish powder mixes consisting of 90% Lake Trout and 10% maize (A) and 10% Lake Trout and 90% maize (B). Cn:x are fatty acids with carbon length n and number of unsaturations x; br are branched-chain acids; IS is internal standard (n-hexatriacontane).

**Fig 6:**
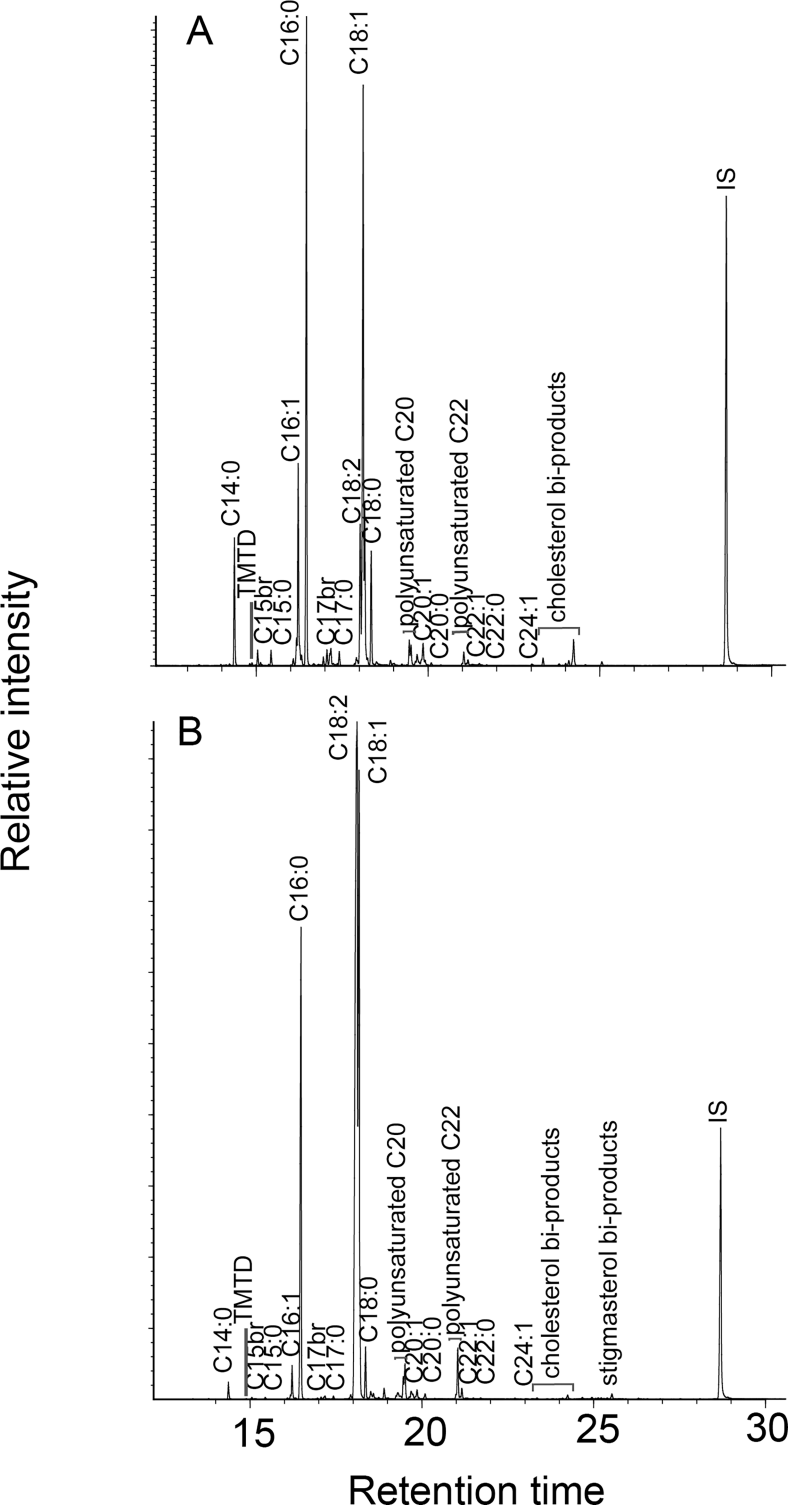
Gas chromatograms of lipid extracts from heated maize-fish powder mixes consisting of 90% Chain Pickerel and 10% maize (A) and 10% Chain Pickerel and 90% maize (B). Cn:x are fatty acids with carbon length n and number of unsaturations x; br are branched-chain acids; APFA Cx are ω‐(o‐alkylphenyl) alkanoic acids with carbon length x.

Certain trends in fatty acid ratios are evident as the proportions of fish in unheated samples diminish, such as a general decrease in C14:0, C16:1, C16:0, C18:0 and C18:1, and an increase in C18:2 (Table 7, Fig 5). The sample containing 50% of Lake Trout consistently deviate from these trends, which we suspect is due to a manipulation error in the lab. Several of these trends, however, are not present in mixes subjected to intense heating (Table 8; Fig 6). Notably, ratios between palmitic and stearic fatty acids (C16:0/C18:0)—often used in the literature to distinguish between plant and animal resources—become ill-suited to distinguish between different categories of foods once samples have been exposed to heat. While the same failure does not apply to other ratios in this study, such as C16:1/C18:1 and (C15:0+C17:0)/C18:0, we maintain that a methodology based on fatty acid ratios alone is unsuitable for identifying the source of archaeological residues. Degradation processes undergone by lipids as a result of use and burial are in part dependent on the types of compounds present and therefore potentially traceable to original vessel contents. However, variation in the circumstances of use (e.g., time, temperature, oxidative conditions) and the nature of the ceramics (e.g., porosity, clay matrix) complicates comparison with experimental analogues. Furthermore, a range of post-burial chemical and enzymatic processes, not fully replicated in our experiment where sherds were only heated, also affect the distribution of lipids in a residue. Such conditions are hard to simulate even through burial experiments. Therefore, we contend that the biomarker approach and the criteria described in [27,28,41] provide the only robust methodology for aquatic identification in archaeological pottery, supplemented where possible by carbon isotope measurements of individual fatty acids.

**Table 7:** Lake Trout and maize unheated powder mixture lipid and biomarker results. Cx:y = fatty acids with carbon length x and number of unsaturations y (C18:1s and C18:2s are the sum of all isomers); br = branched chain acids; TMTD = 4,8,12‐trimethyltridecanoic acid, chol = cholesterol, stig = stigmasterol.

		Percentages of Lake Trout								
	Raw resource based on weight	90.0	80.0	70.0	60.0	50.0	40.0	30.0	20.0	10.0
	Residue based on δ^13^C^a^	96.3	90.1	80.0	72.0	47.4	57.7	46.6	37.0	17.3
	Residue based on FMC^a^	98.7	99.6	76.2	73.5	57.6	55.6	45.1	38.4	18.0
Fatty Acids (relative %)	C14:0	4.96	4.13	3.40	2.64	4.09	1.73	1.39	0.93	0.47
	C15br	0.57	0.67	0.55	0.31	0.45	0.21	0.16	0.10	0.04
	C15:0	0.60	0.52	0.43	0.32	0.43	0.21	0.17	0.11	0.06
	C16br	0.34	0.31	0.31	0.23	trace	0.17	0.15	0.09	0.00
	C16:1	10.52	9.04	7.50	5.84	8.76	4.08	3.13	2.28	1.12
	C16:0	28.20	25.39	21.45	20.74	22.56	17.09	16.86	16.30	15.84
	C17br	1.03	0.63	0.48	1.10	1.77	0.73	0.20	0.41	0.21
	C17:1	0.79	1.13	0.92	0.00	0.00	0.00	0.00	0.00	0.00
	C17:0	0.59	0.56	0.43	0.36	0.52	0.24	0.20	0.16	0.09
	C18:3	0.78	1.01	1.19	3.15	4.13	1.25	1.29	1.37	1.83
	C18:2s	6.22	9.46	14.44	19.65	3.39	28.93	35.29	40.99	47.68
	C18:1s	33.77	34.12	30.86	30.57	32.18	27.30	26.98	25.76	24.57
	C18:0	4.79	4.63	3.45	3.19	4.59	2.38	2.24	1.95	1.62
	C19:0	trace	trace	trace	trace	trace	trace	trace	0.05	0.00
	C20:3	1.93	2.23	4.37	3.05	4.56	4.27	4.12	3.96	3.20
	C20:2	0.59	0.87	1.14	1.02	1.45	0.96	0.88	0.10	trace
	C20:1	1.02	1.79	1.59	0.87	1.27	0.91	0.76	0.56	0.45
	C20:0	0.71	0.16	0.15	trace	trace	0.17	0.18	0.16	0.21
	C22:2	0.27	1.33	4.81	5.93	8.67	8.38	5.12	4.03	2.11
	C22:1	trace	0.20	0.24	0.00	0.00	0.00	0.00	0.00	0.00
	C22:0	trace	trace	trace	trace	0.00	trace	trace	trace	trace
	C24:1	trace	0.17	0.15	trace	0.00	trace	trace	trace	trace
	C24:0	0.00	0.00	0.00	0.00	0.00	trace	trace	trace	trace
Biomarkers		TMTD	TMTD	TMTD	TMTD	TMTD	TMTD	TMTD	TMTD	TMTD
		chol	chol	chol	chol	chol	chol	chol	chol	chol
							stig	stig	stig	stig

^a^Calculated with mass balance equation.

**Table 8:** Chain Pickerel and maize heated powder mixture lipid and biomarker results. Cx:y = fatty acids with carbon length x and number of unsaturations y (C18:1s and C18:2s are the sum of all isomers); br = branched chain acids; APFA Cx = ω‐(o‐alkylphenyl) alkanoic acids with carbon length x; chol = cholesterol.

		Percentages of Chain Pickerel								
	Raw resource based on weight	90.0	80.0	70.0	60.0	50.0	40.0	30.0	20.0	10.0
	Residue based on δ^13^C^a^	91.6	90.4	77.3	70.9	59.2	56.6	34.8	35.8	12.7
	Residue based on FMC^a^	97.6	85.8	70.4	73.8	57.6	68.1	34.3	31.6	9.7
Fatty Acids (relative %)	C14:0	2.66	2.65	1.94	2.02	2.01	1.72	1.65	1.81	2.15
	C15:0	0.89	0.72	0.59	0.51	trace	trace	trace	trace	trace
	C16:1	2.32	2.12	1.99	0.93	0.62	0.63	0.00	0.00	0.00
	C16:0	33.40	33.15	31.73	32.67	29.73	32.16	33.66	35.51	33.53
	C17br	1.84	1.58	1.31	1.27	1.31	1.09	0.44	trace	0.59
	C17:0	1.48	1.28	0.94	1.17	1.43	1.10	1.15	0.96	1.40
	C18:2s	0.00	0.00	0.00	0.39	1.05	0.99	0.90	0.89	1.07
	C18:1s	18.57	21.35	26.75	24.47	21.62	23.39	22.96	25.75	21.27
	C18:0	23.16	20.27	14.29	22.59	33.10	24.82	28.08	24.40	34.56
	C20:1	0.75	0.79	0.77	0.63	0.46	0.73	0.66	0.82	0.82
	C20:0	1.13	1.14	1.03	1.26	1.00	1.52	1.55	1.71	1.92
	C22:0	0.36	0.38	0.49	0.42	0.34	0.48	0.51	0.59	0.43
	C23:0	trace	trace	trace	trace	trace	0.32	trace	trace	trace
	C24:1	1.38	1.20	1.52	0.91	trace	trace	trace	0.00	0.00
	C24:0	0.63	0.47	0.80	0.84	trace	0.66	0.58	0.58	0.00
Biomarkers		TMTD	TMTD	TMTD	TMTD	TMTD	TMTD	TMTD	TMTD	TMTD
		phytanic	phytanic	phytanic	phytanic	phytanic	phytanic	phytanic	phytanic	phytanic
		APFA C18	APFA C18	APFA C18	APFA C18	APFA C18	APFA C18	APFA C18	APFA C18	APFA C18
		APFA C20	APFA C20	APFA C20	APFA C20	APFA C20	APFA C20	APFA C20	APFA C20	APFA C20
		chol	chol	chol	chol	chol	chol	chol		

^a^Calculated with mass balance equation.

The presence of ω-(o-alkylphenyl)alkanoic acids with 18 and 20 carbon atoms, together with at least one of the three isoprenoid fatty acids (phytanic, pristanic or 4,8,12-tetramethyltridecanoic acid) have been established in the literature as the full set of molecular criteria needed for the identification of degraded aquatic products in archaeological residues [27,28]. These biomarkers are routinely recovered from prehistoric encrusted charred cooking residues (e.g., [19–23]). Isoprenoid alkanoic acids (phytanic, pristanic or 4,8,12-TMTD) are at high concentration in freshwater and marine organisms, and positional alisomers of ω-(o-alkylphenyl)alkanoic acids with 16–22 carbon atoms are produced through the protracted heating of polyunsaturated fatty acids present in aquatic organisms at temperatures of at least 270°C [28]. Polyunsaturated fatty acids degrade easily and are thus unlikely to survive in organic residues from archaeological pottery. However, clays can act as an acid or base catalyzing agent and thereby promote the isomerization of double bonds involved in the formation of ω-(o-alkylphenyl)alkanoic acids [27,28]. The latter are more stable compounds and offer a reliable means of detecting the processing of commodities containing unsaturated fatty acids. Because vegetable oils are also rich in C18 triunsaturated alkanoic acids, only residues containing both the C18 and C20 ω-(o-alkylphenyl)alkanoic acids are indicators of aquatic lipid residues.

**Fig 7:**
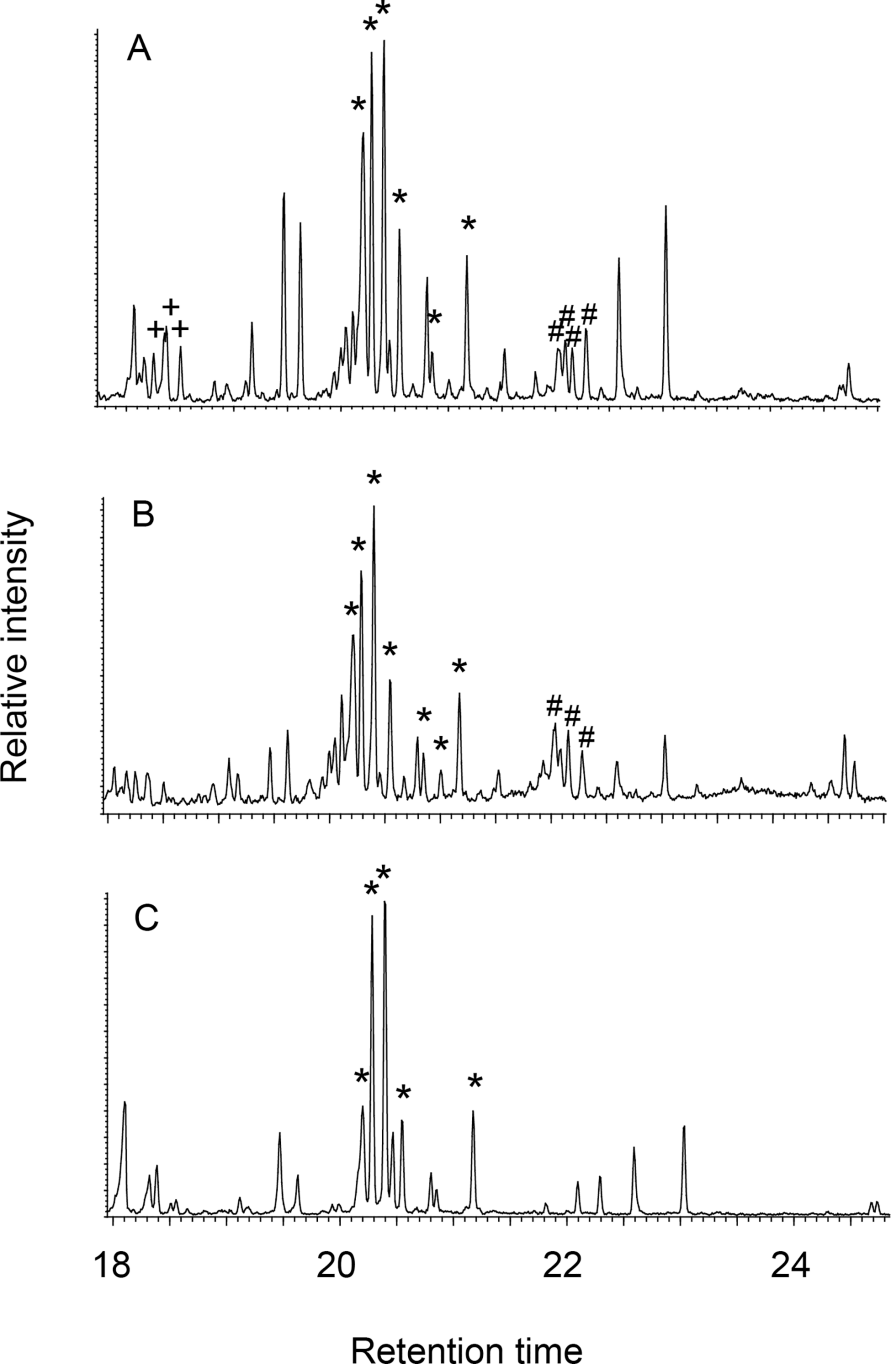
Partial m/z 105 ion chromatograms showing ω-(o-alkylphenyl)alkanoicacids with 16(+), 18(*), and 20(#) carbon atoms in heated samples containing lake trout (A), chain pickerel (B) and maize (C).

In this experiment, we were able to confirm the formation of ω-(o-alkylphenyl)alkanoic acids with 16–20 carbon atoms when food sources containing unsaturated fatty acids are exposed to prolonged and intense heating (270°C for 17 hours) in the presence of a clay matrix. Analysis of single ingredients (e.g., dried maize, dried Chain Pickerel, and dried Lake Trout) confirmed that degraded aquatic and plant oils cannot be distinguished based on the presence of ω-(o-alkylphenyl)octadecanoic acids alone (Table 9), although Fig 7 suggests that maize and freshwater fish may contain varying proportions of different ω-(o-alkylphenyl)octadecanoic acid isomers, defined by the length of the alkyl side chain.

**Table 9:** Single resource heated lipid and biomarker results. Cx:y = fatty acids with carbon length x and number of unsaturations y (C18:1s and C18:2s are the sum of all isomers); br = branched chain acids; TMTD = 4,8,12‐trimethyltridecanoic acid; APFA Cx = ω‐(o‐alkylphenyl) alkanoic acids with carbon length x.

	Compounds	Maize	Chain Pickerel	Lake Trout
Fatty Acids (relative %)	C12	0.00	0.00	trace
	C13	0.00	0.00	trace
	C14	0.00	1.93	4.76
	C15br	0.00	0.70	2.03
	C15	0.00	4.95	8.79
	C16:1	0.00	2.60	3.11
	C16	38.41	27.87	29.43
	C17br	0.00	2.72	3.82
	C17	0.00	1.49	1.86
	C18:2s	1.98	0.00	0.00
	C18:1s	22.99	16.15	18.77
	C18	12.26	13.38	12.80
	C20:1	0.00	1.31	2.53
	C20	3.74	0.75	1.15
	C22:1	0.00	trace	0.55
	C22	3.12	trace	0.40
	C23	0.00	0.00	trace
	C24:1	0.00	2.95	0.79
	C24	0.00	0.63	trace
Biomarkers			TMTD	TMTD
			phytanic	phytanic
		APFA C18	APFA C18	APFA C18
			APFA C20	APFA C20
			Chol	Chol

Significantly, the complete set of aquatic biomarkers, specifically ω-(o-alkylphenyl)alkanoic acids with 18 and 20 carbon atoms and two isoprenoid alkanoic acids (phytanic acid and 4,8,12-tetramethyltridecanoic acid) were detected in all heated maize-fish powder samples. Interestingly, 4,8,12-TMTD was also detected in all unheated Lake Trout-maize samples, even when the raw fish represented as little as 10% of the mixture. Cholesterol, sometimes in combination with its oxidation products, was also identified in all unheated Lake Trout-maize powder mixes, which also contain stigmasterol when maize composed over 60% of the mixture. In heated samples, however, cholesterol bi-products were not detected when raw Chain Pickerel represented less than 30% of the mixture.

In sum, in this experiment the full set of aquatic biomarkers (i.e., ω-(o-alkylphenyl)alkanoic acids with 18 and 20 carbon atoms two isoprenoid fatty acids) was present in all samples, even when raw fish represented as little as 10% of the mixture. Of note is that our results suggest that these biomarkers may be present in a residue without a statistically significant FRO.

## Discussion and conclusions

Potential problems with ^14^C ages on charred cooking residues encrusted on pottery resulting from the presence of ancient carbon from aquatic resources has drawn considerable attention over the last decade and a half. This has been particularly true in northern Europe but has also been identified as a potential problem in other regions [42]. Most often concerns are raised when ^14^C ages obtained on residues are older than those obtained on terrestrial resource remains recovered from the same archaeological contexts as the radiocarbon-dated residues. A more parsimonious methodology would be to determine if C from fish is present in residues where the possibility of an FRE for the location and time in question has been established through independent lines of evidence (e.g., dates on fish remains, lake sediment analyses). Laboratory work has identified specific biomarkers for aquatic resources that can be extracted from absorbed and encrusted residues [6]. However, empirical work has not been done to establish how much fish needed to have been cooked in a pot to contribute sufficient ancient carbon to residue formation to produce statistically significant FROs and result in the presence of aquatic biomarkers. Our goals here were to provide preliminary assessments of these issues.

Our results indicate that there is a very high positive correlation between the percentage of C from fish in residues and FROs. However, there is no such direct relationship between the fraction of raw fish cooked in a pot and the fraction of fish C in the residue. Statistically significant FROs may occur when fish constitute <1% of the raw resource mix, but may also not occur until fish represent over 90% of raw resources depending on the resource(s) with which it was cooked and the size of the ^14^C age error. Our results also indicate that the complete sets of biomarkers, in this case the presence of ω-(o-alkylphenyl)alkanoic acids with 18 and 20 carbon atoms and phytanic acid, may be detected when fish C contributes little to residue formation. Thus, it is possible for aquatic biomarkers to be identified in a residue in the absence of a statistically significant FRO when a freshwater reservoir effect was present. This in turn emphasizes the need to assess the potential for ancient carbon reservoirs for specific periods of time in question prior to considering charred, encrusted residues for radiocarbon dating. Contemporary water chemistry and aquatic organisms are not adequate analogues for prehistoric reservoirs because modern land practices have significant effects on freshwater reservoirs [43].

Moreover, our results demonstrate compound-specific^14^C analysis (CSRA) could also be applied to issues surrounding FROs from charred cooking residues. To date, applications of CSRA to pottery residues have targeted C16:0 and C18:0 fatty acids, which typically are the most abundant fatty acids preserved in potsherds [4,5,44]. Recent advances in preparative gas chromatography (PGC), however, have decreased sample size requirements and opened the door to dating and comparing ages associated with a wider range of discrete biomarkers [45].
