# Correction: Freshwater reservoir offsets and food crusts: Isotope, AMS, and lipid analyses of experimental cooking residues

**DOI:** 10.1371/journal.pone.0197722

**Published:** 2018-05-22

**Authors:** John P. Hart, Karine Taché, William A. Lovis

The images for Figs 5 and 6 are incorrectly switched. The image that appears as Fig 5 should be Fig 6, and the image that appears as Fig 6 should be Fig 5. The figure captions appear in the correct order.

**Fig 5:**
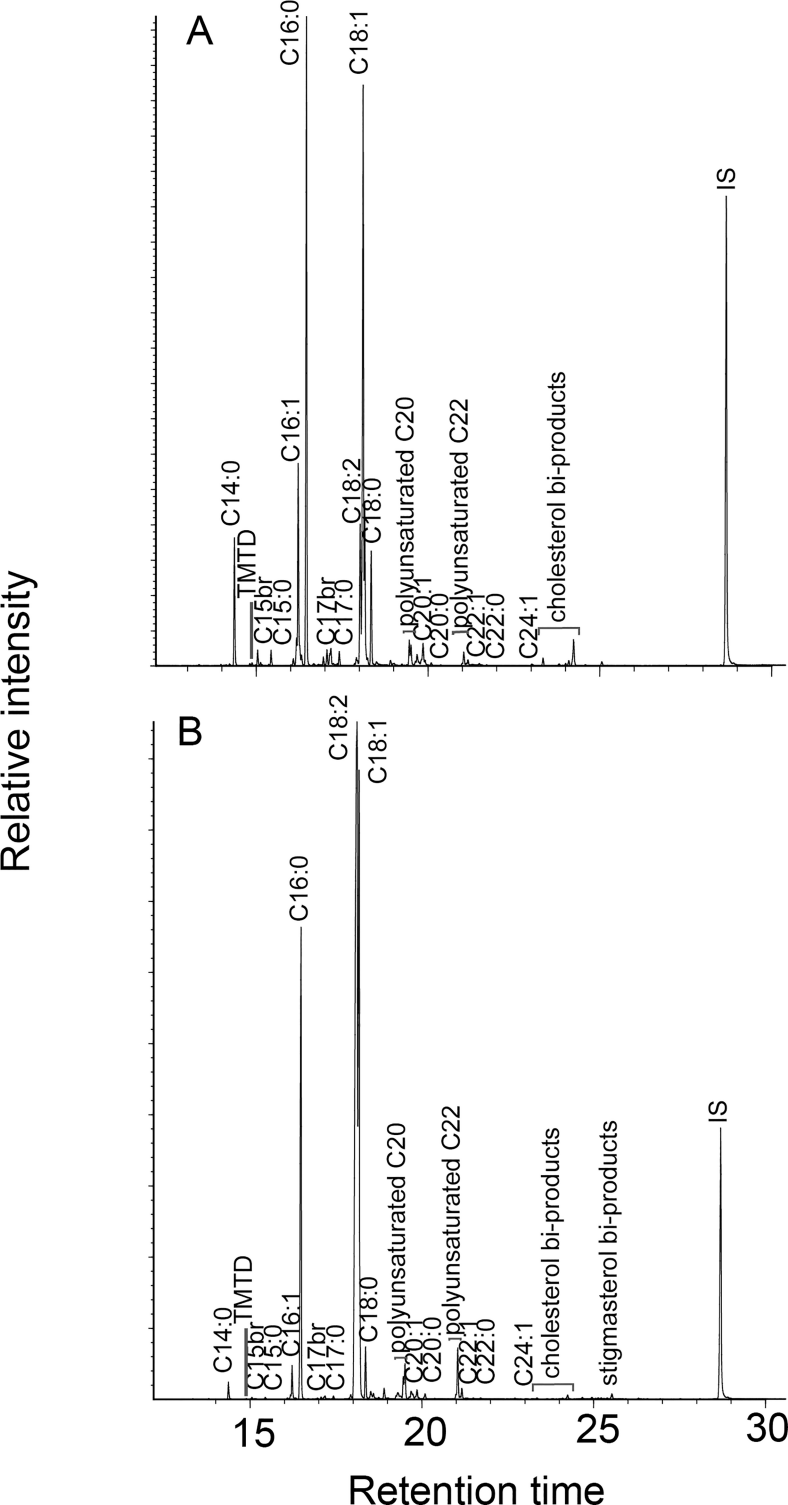
Gas chromatograms of lipid extracts from unheated maize-fish powder mixes consisting of 90% Lake Trout and 10% maize (A) and 10% Lake Trout and 90% maize (B). Cn:x are fatty acids with carbon length n and number of unsaturations x; br are branched-chain acids; IS is internal standard (n-hexatriacontane).

**Fig 6:**
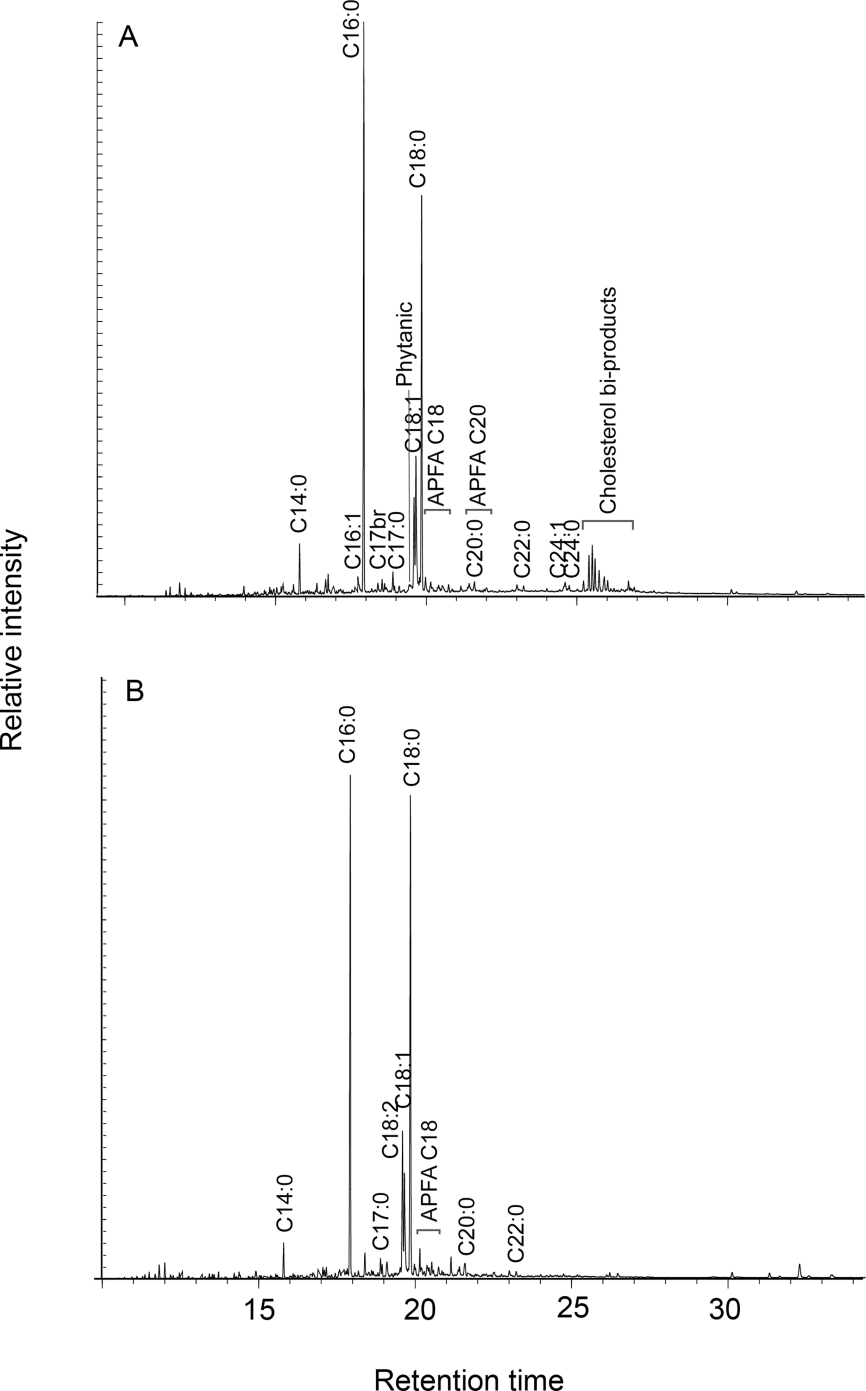
Gas chromatograms of lipid extracts from heated maize-fish powder mixes consisting of 90% Chain Pickerel and 10% maize (A) and 10% Chain Pickerel and 90% maize (B). Cn:x are fatty acids with carbon length n and number of unsaturations x; br are branched-chain acids; APFA Cx are ω‐(o‐alkylphenyl) alkanoic acids with carbon length x.
